# Crystal structures and hydrogen bonding in the isotypic series of hydrated alkali metal (K, Rb and Cs) complexes with 4-amino­phenyl­arsonic acid

**DOI:** 10.1107/S2056989017000445

**Published:** 2017-01-17

**Authors:** Graham Smith, Urs D. Wermuth

**Affiliations:** a Science and Engineering Faculty, Queensland University of Technology, GPO Box 2434, Brisbane, Queensland 4001, Australia; b School of Natural Sciences, Griffith University, Nathan, Queensland 4111, Australia

**Keywords:** crystal structure, *p*-arsanilic acid, coordination polymers, alkali metal salts, hydrogen bonding

## Abstract

The structures of the alkali metal (K, Rb and Cs) complex salts with 4-amino­phenyl­arsonic acid (*p*-arsanilic acid) manifest an isotypic series with the general formula [*M*_2_(C_6_H_7_AsNO_3_)_2_(H_2_O)_3_], with *M* = K {poly[di-μ_3_-4-amino­phenyl­arsonato-tri-μ_2_-aqua-dipotassium], [K_2_(C_6_H_7_AsNO_3_)_2_(H_2_O)_3_], (I)}, Rb {poly[di-μ_3_-4-amino­phenyl­arsonato-tri-μ_2_-aqua-dirubidium], [Rb_2_(C_6_H_7_AsNO_3_)_2_(H_2_O)_3_], (II)}, and Cs {poly[di-μ_3_-4-amino­phenyl­arsonato-tri-μ_2_-aqua-dirubidium], [Cs_2_(C_6_H_7_AsNO_3_)_2_(H_2_O)_3_], (III)}, in which the repeating structural units lie across crystallographic mirror planes containing two independent and different metal cations and a bridging water mol­ecule, with the two hydrogen *p*-arsanilate ligands and the second water mol­ecule lying outside the mirror plane. The bonding about the two metal cations in all complexes is similar, one five-coordinate, the other progressing from five-coordinate in (I) to eight-coordinate in both (II) and (III), with overall *M*—O bond-length ranges of 2.694 (5)–3.009 (7) (K), 2.818 (4)–3.246 (4) (Rb) and 2.961 (9)–3.400 (10) Å (Cs). The additional three bonds in (II) and (III) are the result of inter-metal bridging through the water ligands. Two-dimensional coordination polymeric structures with the layers lying parallel to (100) are generated through a number of bridging bonds involving the water mol­ecules (including hydrogen-bonding inter­actions), as well as through the arsanilate O atoms. These layers are linked across [100] through amine N—H⋯O hydrogen bonds to arsonate and water O-atom acceptors, giving overall three-dimensional network structures.

## Abstract 2

*p*-Arsanilic acid forms an isotypic set of three compounds with the alkali metals K, Rb and Cs, in which the primary layered coordination polymeric structures have similar asymmetric units comprising two independent and different metal cations and a bridging water mol­ecule which lie within crystallographic mirror planes parallel to (100). The layers are linked across [100] through amine N—H⋯O hydrogen bonds to arsonate and water O-atom acceptors, giving overall three-dimensional network structures.

## Chemical context   

Arsenical 4-amino­phenyl­arsonic acid (*p*-arsanilic acid) has biological significance as an anti-helminth in veterinary applications (Steverding, 2010 ▸; O’Neil, 2001 ▸) and as a hydrated sodium salt (atox­yl) that had early usage as an anti-syphilitic (Ehrlich & Bertheim, 1907 ▸; Bosch & Rosich, 2008 ▸). The crystal structure of this salt has been determined together with the NH_4_^+^ salt (Smith & Wermuth, 2014 ▸); the structure of the parent *p*-arsanilic acid, which exists as a zwitterion, is also known (Shimada, 1961 ▸; Nuttall & Hunter, 1996 ▸). We have also determined the structures of the alkaline earth metal (Mg, Ca, Sr, Ba) salts of the acid (Smith & Wermuth, 2017 ▸). However, simple *p*-arsanilate single-metal complex structures are not common in the Cambridge Structure Database (Groom *et al.*, 2016 ▸), examples being with Ag^I^ (three forms), Zn, Pb and Cd (Lesikar-Parrish *et al.*, 2013 ▸; Xiao *et al.*, 2015 ▸); Zn (Lin *et al.*, 2012 ▸); Cd (Liu *et al.*, 2010 ▸); Sn^IV^ (Xie *et al.*, 2008 ▸); V^IV^ and V^V^ (Breen *et al.*, 2012 ▸; Chen *et al.*, 2012 ▸; Khan *et al.*, 1992 ▸); UO_2_ (Adelani *et al.*, 2012 ▸). Mixed-metal and/or mixed-ligand complexes are common, *e.g.* Co^II^/Mo=O, Ni^II^/Mo=O, Cu^II^/Mo=O and Zn/Mo=O with *p*-arsanilate and ligands such as 2,2′-bi­pyridine, 4,4′-bi­pyridine and 1,10-phenanthroline (Smith *et al.*, 2013 ▸).
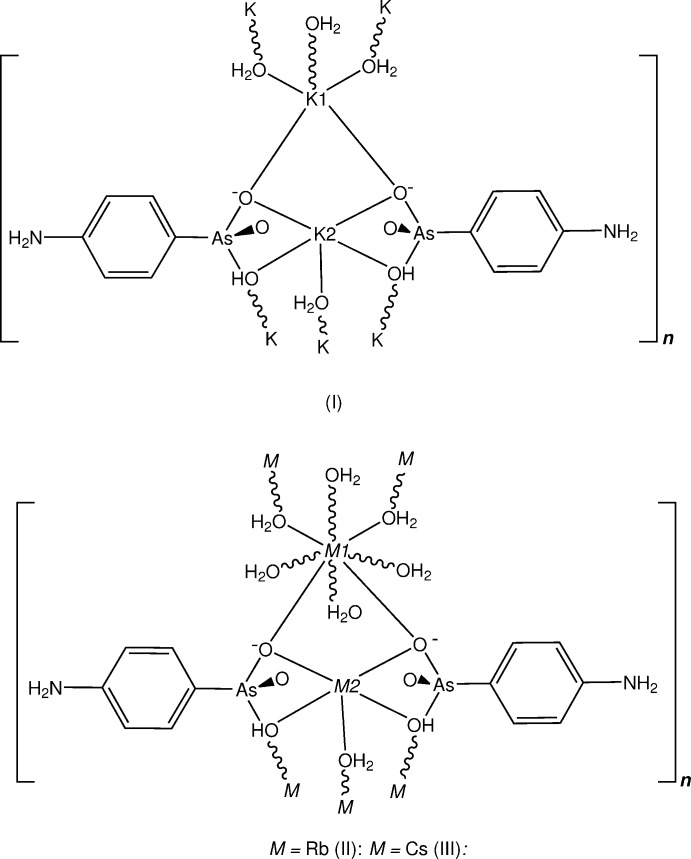


In an attempt to complete the structures of the alkali metal series of *p*-arsanilate salts, our reaction of the acid with potassium carbonate, rubidium carbonate and caesium carbonate in ethanol/water resulted in the formation of the crystalline hydrated salts with general formula [*M* ^+^_2_(C_6_H_7_AsNO_3_)^−^_2_·3H_2_O]. Compounds (I) (*M* = K), (II) (Rb) and (III) (Cs) and their crystal structures are reported herein. However, suitable crystals of the Li analogue were not obtained to allow its crystal structure determination.

## Structural commentary   

**Figure 1:**
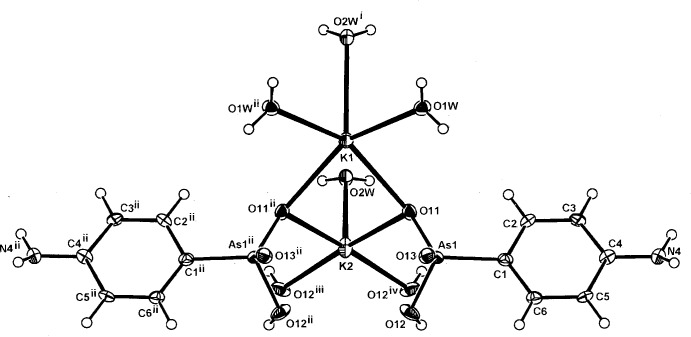
The mol­ecular configuration and atom numbering scheme for the complex unit in (I). The metal cations (K1 and K2) and the water mol­ecule (O2*W*) lie on a mirror plane with mirror-related atoms indicated by symmetry code (ii) −*x* + 1, −*y*, *z* + 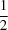. For other codes, see Table 1 ▸. Non-H atoms are shown as displacement ellipsoids at the 40% probability level.

The structures of the three title compounds [(I), (II) and (III)] form an isotypic series, with the asymmetric units in each comprising two independent and different metal complex cations (*M*1 and *M*2), which lie on crystallographic mirror planes that also contain one of the coordinating water mol­ecules (O2*W*), with the hydrogen *p*-arsanilate ligands and the second water mol­ecules (O*W*1, O1*W* ^ii^) [symmetry code: (ii) −*x* + 1, −*y*, *z*] lying across the mirror plane (Figs. 1 ▸, 2 ▸ and 3 ▸, respectively). In all three examples, the *M*2 cation is five-coordinate, while with *M*1, the coordination spheres progress from five-coordinate in (I) to eight-coordinate in (II) and (III). The overall *M*—O bond length ranges are 2.694 (5)–3.009 (7) Å (K) (Table 1 ▸), 2.818 (4)–3.246 (4) Å (Rb) (Table 2 ▸) and 2.961 (9)–3.400 (10) Å (Cs) (Table 2 ▸). The amine N atom is not involved in bonding to the metal, as is the case in a number of other *p*-arsanilate complexes, *e.g*. with Zn (Lin *et al.*, 2012 ▸). The *M*1O_5_ polyhedra in all three structures comprise four bridging arsonate O atoms and the *μ*_2_ bridging water mol­ecule (O2*W*) (Tables 1 ▸, 2 ▸ and 3 ▸). The second *M*2O_5_ polyhedron in (I) comprises the bridging O11 and O11^ii^ donors, the *μ*_2_-O2*W* ^i^ [symmetry code: (i) −*x* + 1, −*y* + 2, *z* + 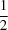] donor and two monodentate water mol­ecules (O1*W* and O1*W* ^i^) (Table 1 ▸).

With (II) and (III), the irregular *M*2O_8_ coordination sphere comprises all bonds mentioned in the description of the K complex (I), and in addition, the Rb and Cs bond length expansion allows further coordination sites through additional bridging bonds to both of the water mol­ecules (two through O1*W* and one through O2*W*), (Tables 2 ▸ and 3 ▸). The *M*1⋯*M*2 separations are 4.139 (3) Å [for (I)], 4.2500 (11) Å [for (II)] and 4.3498 (15) Å [for (III)]. There are also slightly shorter *M*1⋯*M*1^i^ separations in all structures: 4.079 (3) Å (I), 4.1953 (13) Å (II) and 4.3127 (16) Å (III). Relatively short *M*2⋯As1 separations are present within the repeat unit in all three structures: 3.6369 (19) Å (I), 3.7796 (8) Å (II) and 3.9488 (14) Å (III).

**Figure 4:**
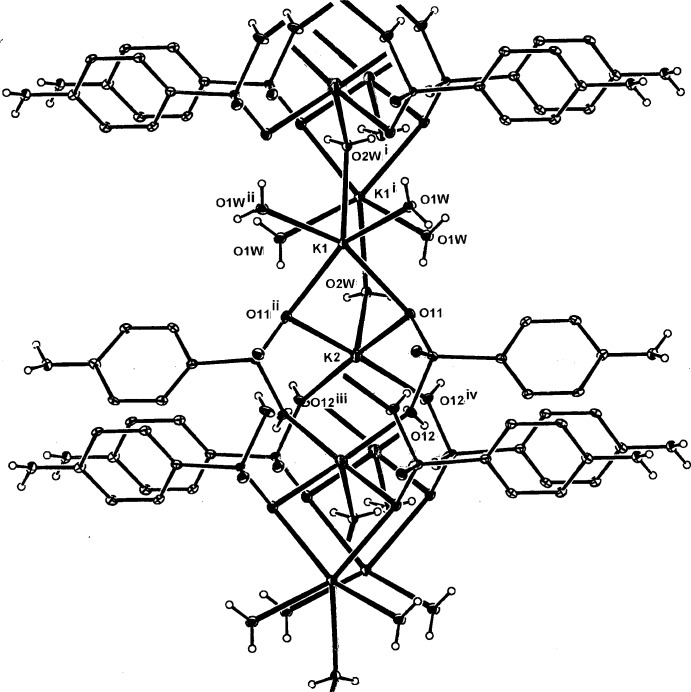
A partial expansion of the two-dimensional coordination polymeric sheet structure of (I), which extends across the mirror plane parallel to (100). Aromatic H atoms are omitted. For symmetry codes, see Table 1 ▸.

**Figure 5:**
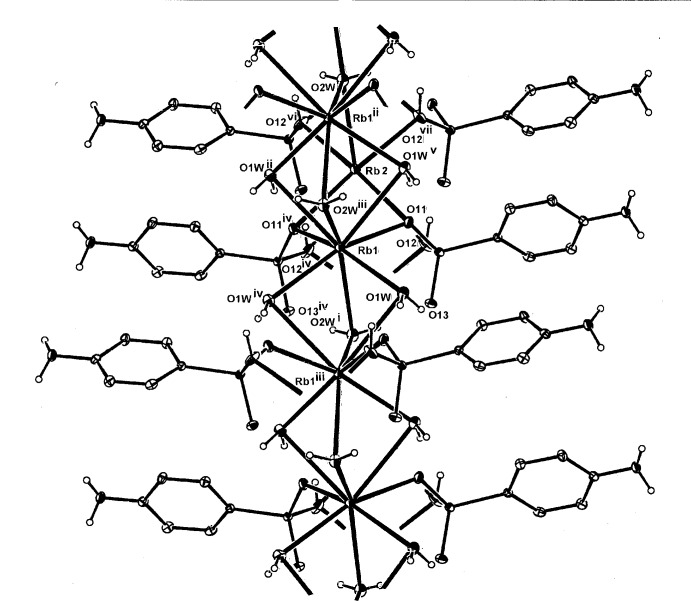
A partial expansion of the two-dimensional coordination polymeric sheet structure of (II) [or (III)], which extends across the mirror plane parallel to (100). Aromatic H atoms have been omitted.

In all structures, two-dimensional coordination polymeric complex structures are generated, with the layers lying in the mirror planes parallel to (100). Fig. 4 ▸ shows the basic makeup of the layer in (I) while those for (II) or (III) are shown in Fig. 5 ▸. The water mol­ecule O2*W* provides hydrogen-bonding links across the mirror plane to arsonate O13 acceptors (Tables 4 ▸, 5 ▸ and 6 ▸).

## Supra­molecular features   

**Figure 6:**
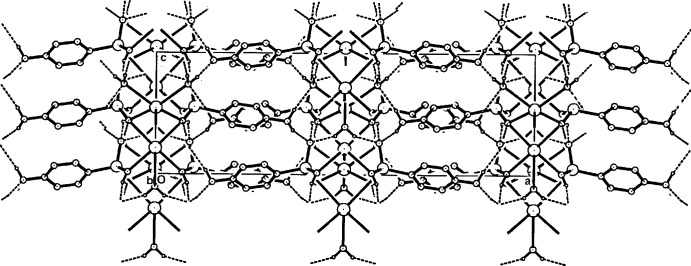
A view of the packing in the unit cell of (I) along [010], showing the associated cation/anion sheets linked peripherally across [100] by hydrogen bonds involving the anilinium amine groups. Hydrogen-bonding inter­actions are shown as dashed lines and aromatic H atoms have been omitted.

In the crystals of all three compounds, similar overall packing modes are observed, with the coordination polymeric layers lying along the mirror planes inter-linked across [100] through amine N4—H⋯O hydrogen bonds to arsonate O13 and water O1*W* acceptors (Tables 4 ▸, 5 ▸ and 6 ▸). In this respect, they resemble the crystal packing of the Na *p*-arsanilate analogue (Smith & Wermuth, 2014 ▸) but the structure of that compound (a trihydrate) differs from the current isotypic set in having significantly different coordination spheres, also lacking the mirror symmetry of the primary polymeric layers in (I)–(III). With these, the N4 amino group acts as an acceptor to an O1*W* hydrogen bond. The water mol­ecule O1*W* also forms a hydrogen bond with O11^vi^ [symmetry code: (vi) *x*, −*y* + 2, *z* + 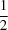] in (I), but not in (II) or (III). The protonated *p*-arsanilate O atom (O12) forms an intra-layer hydrogen bond with an O11 acceptor, giving overall three-dimensional network structures in all cases (Figs. 6 ▸ and 7 ▸). No π–π associations are present in the structures.

## Database survey   

Three-dimensional supra­molecular structures involving complexes of hydrogen *p*-arsanilate and mixed metal types, as distinct from those involving uni-metal types, such as in (I)–(III) and in those examples which have been previously mentioned in the *Chemical context* section of this article, are worthy of noting here. Mixed-metal-ligand examples (Smith *et al.*, 2013 ▸) as well as mixed–metal structures add to the complexity of the coordination polymeric structures commonly generated, *e.g*. in the Mo/Ag, Mo/Cu and W/Na polyoxidometallate compounds (Johnson *et al.*, 2002 ▸), the Mo/V cage structure (Onet *et al.*, 2011 ▸) or the V/Na structure (Breen & Schmitt, 2008 ▸).

## Synthesis and crystallization   

Compounds (I)–(III) were synthesized by heating together for 5 min, 1 mmol qu­anti­ties of 4-amino­phenyl­arsonic acid and 0.5 mmol of either K_2_CO_3_ [for (I)], Rb_2_CO_3_ [for (II)] or Cs_2_CO_3_ [for (III)], in 20 ml of 50% ethanol/water (*v*/*v*). Room temperature evaporation of the solutions gave colourless crystal plates of the title compounds from which specimens were cleaved for the X-ray analyses.

## Refinement details   

Crystal data, data collection and structure refinement details are summarized in Table 7 ▸. Hydrogen atoms potentially involved in hydrogen-bonding inter­actions were located by difference methods but their positional parameters were restrained in the refinement with N—H = 0.88 Å and O—H = 0.86 Å, and with *U*_iso_(H) = 1.2*U*_eq_(N) or 1.5*U*_eq_(O). Other H atoms were included in the refinement at calculated positions, C—H = 0.95 Å, and treated as riding with *U*_iso_(H) = 1.2*U*_eq_(C).

## Supplementary Material

Crystal structure: contains datablock(s) global, I, II, III. DOI: 10.1107/S2056989017000445/wm5350sup1.cif

Structure factors: contains datablock(s) I. DOI: 10.1107/S2056989017000445/wm5350Isup2.hkl

Structure factors: contains datablock(s) II. DOI: 10.1107/S2056989017000445/wm5350IIsup3.hkl

Structure factors: contains datablock(s) III. DOI: 10.1107/S2056989017000445/wm5350IIIsup4.hkl

CCDC references: 1526425, 1526424, 1526423

Additional supporting information:  crystallographic information; 3D view; checkCIF report

## Figures and Tables

**Figure 2:**
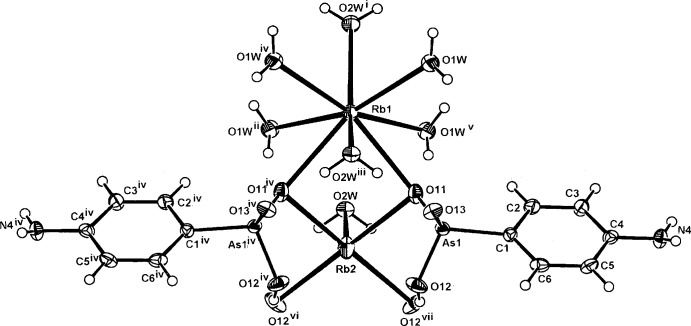
The mol­ecular configuration and atom numbering scheme for the complex unit in the isotypic structure (II). The metal cations (Rb1 and Rb2) and the water mol­ecule (O2*W*) also lie on a mirror plane. For symmetry codes, see Table 2 ▸. Non-H atoms are shown as displacement ellipsoids at the 40% probability level.

**Figure 3:**
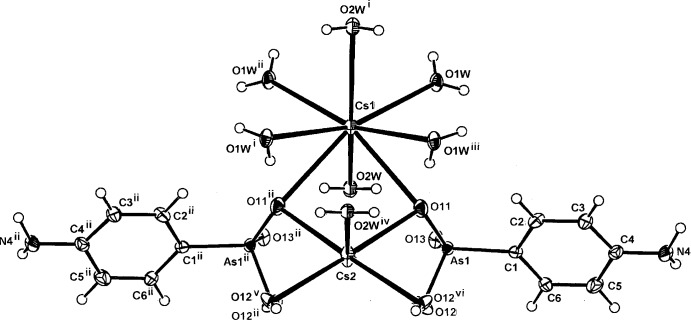
The mol­ecular configuration and atom numbering scheme for the complex unit in the isotypic structure (III). The metal cations (Cs1 and Cs2) and the water mol­ecule (O2*W*) also lie on a mirror plane. For symmetry codes, see Table 3 ▸. Non-H atoms are shown as displacement ellipsoids at the 40% probability level.

**Figure 7:**
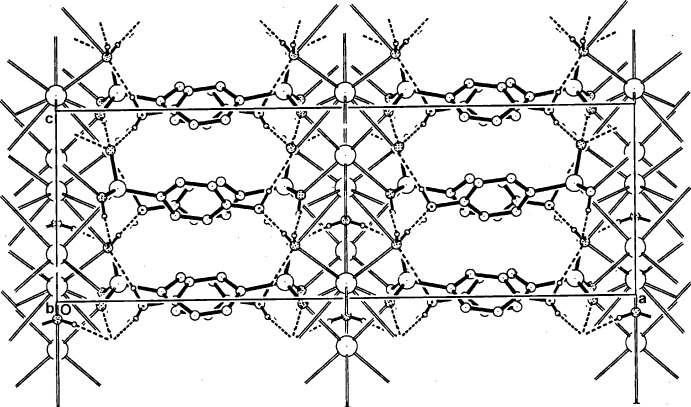
A view of the packing in the unit cell of (II) [or (III)] along [010], showing the associated cation/anion sheets linked peripherally across [100] by hydrogen bonds involving the amine groups.

**Table 1:** Selected bond lengths (Å) for (I)

K1—O1*W*	2.766 (5)	K2—O2*W*	3.009 (7)
K1—O11	2.824 (4)	K2—O11	2.713 (5)
K1—O2*W* ^i^	2.959 (7)	K2—O12^iii^	2.694 (5)
K1—O1*W* ^ii^	2.766 (5)	K2—O11^ii^	2.713 (5)
K1—O11^ii^	2.824 (4)	K2—O12^iv^	2.694 (5)

Symmetry codes: (i) 
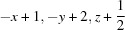
; (ii) 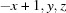; (iii) 
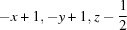
; (iv) 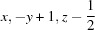.

**Table 2:** Selected bond lengths (Å) for (II)

Rb1—O1*W*	2.917 (4)	Rb1—O1*W* ^v^	3.246 (4)
Rb1—O11	2.925 (3)	Rb2—O2*W*	3.193 (6)
Rb1—O2*W* ^i^	3.151 (6)	Rb2—O11	2.863 (3)
Rb1—O1*W* ^ii^	3.246 (4)	Rb2—O12^vi^	2.818 (4)
Rb1—O2*W* ^iii^	3.109 (5)	Rb2—O11^iv^	2.863 (3)
Rb1—O1*W* ^iv^	2.917 (4)	Rb2—O12^vii^	2.818 (4)
Rb1—O11^iv^	2.925 (3)		

Symmetry codes: (i) 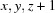; (ii) 
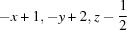
; (iii) 
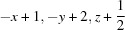
; (iv) 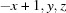; (v) 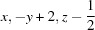; (vi) 
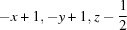
; (vii) 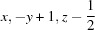.

**Table 3:** Selected bond lengths (Å) for (III)

Cs1—O1*W*	3.087 (9)	Cs1—O1*W* ^iii^	3.400 (10)
Cs1—O2*W*	3.286 (13)	Cs2—O11	3.024 (8)
Cs1—O11	3.040 (8)	Cs2—O2*W* ^iv^	3.324 (13)
Cs1—O1*W* ^i^	3.400 (10)	Cs2—O12^v^	2.961 (9)
Cs1—O2*W* ^i^	3.295 (12)	Cs2—O11^ii^	3.024 (8)
Cs1—O1*W* ^ii^	3.087 (9)	Cs2—O12^vi^	2.961 (9)
Cs1—O11^ii^	3.040 (8)		

Symmetry codes: (i) 
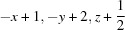
; (ii) 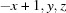; (iii) 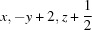; (iv) 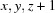; (v) 
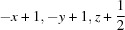
; (vi) 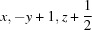.

**Table 4:** Hydrogen-bond geometry (Å, °) for (I)

*D*—H⋯*A*	*D*—H	H⋯*A*	*D*⋯*A*	*D*—H⋯*A*
O1*W*—H11*W*⋯N4^v^	0.88 (6)	2.05 (6)	2.915 (7)	171 (5)
O1*W*—H12*W*⋯O11^vi^	0.89 (5)	1.77 (5)	2.660 (6)	175 (7)
O2*W*—H21*W*⋯O13^vii^	0.86 (5)	2.09 (6)	2.819 (6)	142 (6)
O12—H12⋯O13^iv^	0.87 (6)	1.70 (6)	2.538 (7)	160 (7)
N4—H41⋯O1*W* ^viii^	0.86 (5)	2.17 (5)	3.010 (7)	164 (5)
N4—H42⋯O13^ix^	0.87 (6)	2.15 (6)	2.984 (7)	160 (5)

Symmetry codes: (iv) 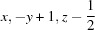; (v) 
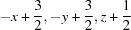
; (vi) 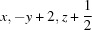; (vii) 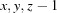; (viii) 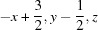; (ix) 
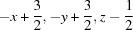
.

**Table 5:** Hydrogen-bond geometry (Å, °) for (II)

*D*—H⋯*A*	*D*—H	H⋯*A*	*D*⋯*A*	*D*—H⋯*A*
O1*W*—H11*W*⋯N4^viii^	0.89 (5)	2.04 (4)	2.923 (6)	176 (5)
O2*W*—H21*W*⋯O13^ix^	0.88 (4)	1.97 (4)	2.852 (5)	173 (5)
O12—H12⋯O13^vii^	0.86 (3)	1.73 (4)	2.552 (5)	158 (5)
N4—H41⋯O13^x^	0.86 (5)	2.18 (4)	3.022 (5)	167 (4)
N4—H42⋯O1*W* ^xi^	0.88 (4)	2.13 (4)	3.005 (6)	176 (4)

Symmetry codes: (vii) 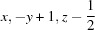; (viii) 
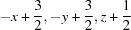
; (ix) 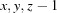; (x) 
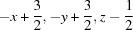
; (xi) 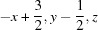.

**Table 6:** Hydrogen-bond geometry (Å, °) for (III)

*D*—H⋯*A*	*D*—H	H⋯*A*	*D*⋯*A*	*D*—H⋯*A*
O1*W*—H11*W*⋯N4^vii^	0.89 (9)	2.28 (13)	2.952 (13)	132 (10)
O2*W*—H21*W*⋯O13	0.89 (12)	2.16 (12)	2.850 (10)	134 (12)
O12—H12⋯O13^vi^	0.88 (5)	2.00 (12)	2.567 (13)	121 (12)
N4—H41⋯O1*W* ^viii^	0.83 (14)	2.12 (15)	2.928 (15)	164 (9)
N4—H42⋯O13^ix^	0.91 (14)	2.20 (14)	3.082 (13)	166 (15)

Symmetry codes: (vi) 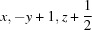; (vii) 
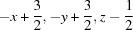
; (viii) 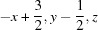; (ix) 
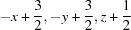
.

**Table 7:** Experimental details

	(I)	(II)	(III)
Crystal data			
Chemical formula	[K_2_(C_6_H_7_AsNO_3_)_2_(H_2_O)_3_]	[Rb_2_(C_6_H_7_AsNO_3_)_2_(H_2_O)_3_]	[Cs_2_(C_6_H_7_AsNO_3_)_2_(H_2_O)_3_]
*M*_r_	564.34	657.08	751.96
Crystal system, space group	Orthorhombic, *C* *m* *c*2_1_	Orthorhombic, *C* *m* *c*2_1_	Orthorhombic, *C* *m* *c*2_1_
Temperature (K)	200	200	200
*a*, *b*, *c* (Å)	24.3426 (18), 10.4266 (7), 7.8315 (6)	24.4783 (19), 10.4577 (9), 8.0978 (7)	24.650 (3), 10.4373 (9), 8.3992 (7)
*V* (Å^3^)	1987.7 (3)	2072.9 (3)	2160.9 (4)
*Z*	4	4	4
Radiation type	Mo *K*α	Mo *K*α	Mo *K*α
μ (mm^−1^)	3.83	7.94	6.46
Crystal size (mm)	0.35 × 0.22 × 0.11	0.35 × 0.20 × 0.12	0.40 × 0.22 × 0.10

Data collection			
Diffractometer	Oxford Diffraction Gemini-S CCD-detector	Oxford Diffraction Gemini-S CCD-detector	Oxford Diffraction Gemini-S CCD-detector
Absorption correction	Multi-scan (*CrysAlis PRO*; Rigaku OD, 2015 ▸)	Multi-scan (*CrysAlis PRO*; Rigaku OD, 2015 ▸)	Multi-scan (*CrysAlis PRO*; Rigaku OD, 2015 ▸)
*T*_min_, *T*_max_	0.650, 0.980	0.375, 0.980	0.217, 0.980
No. of measured, independent and observed [*I* > 2σ(*I*)] reflections	2874, 1834, 1710	3623, 2093, 1899	4941, 1883, 1787
*R*_int_	0.020	0.026	0.030
(sin θ/λ)_max_ (Å^−1^)	0.688	0.683	0.687

Refinement			
*R*[*F* ^2^ > 2σ(*F* ^2^)], *wR*(*F* ^2^), *S*	0.030, 0.125, 1.23	0.032, 0.072, 1.03	0.041, 0.160, 1.18
No. of reflections	1834	2093	1883
No. of parameters	145	146	145
No. of restraints	8	8	8
H-atom treatment	H atoms treated by a mixture of independent and constrained refinement	H atoms treated by a mixture of independent and constrained refinement	H atoms treated by a mixture of independent and constrained refinement
Δρ_max_, Δρ_min_ (e Å^−3^)	0.50, −0.84	0.70, −0.46	1.69, −1.00
Absolute structure	Flack (1983 ▸), 1281 Friedel pairs	Flack (1983 ▸), 1309 Friedel pairs	Flack (1983 ▸), 1405 Friedel pairs
Absolute structure parameter	0.03 (2)	−0.008 (12)	0.10 (4)

Computer programs: *CrysAlis PRO* (Rigaku OD, 2015 ▸), *SIR92* (Altomare *et al.*, 1993 ▸), *SHELXL97* (Sheldrick, 2008 ▸) within *WinGX* (Farrugia, 2012 ▸) and *PLATON* (Spek, 2009 ▸).

## supplementary crystallographic information

### (I) Poly[di-µ3-4-aminophenylarsonato-tri-µ2-aqua-dipotassium] Crystal data

**Table:**

[K_2_(C_6_H_7_AsNO_3_)_2_(H_2_O)_3_]	*F*(000) = 1128
*M**_r_* = 564.34	*D*_x_ = 1.886 Mg m^−^^3^
Orthorhombic, *C**m**c*2_1_	Mo *K*α radiation, λ = 0.71073 Å
Hall symbol: C 2c -2	Cell parameters from 1421 reflections
*a* = 24.3426 (18) Å	θ = 4.1–28.7°
*b* = 10.4266 (7) Å	µ = 3.83 mm^−^^1^
*c* = 7.8315 (6) Å	*T* = 200 K
*V* = 1987.7 (3) Å^3^	Prism, colourless
*Z* = 4	0.35 × 0.22 × 0.11 mm

### (I) Poly[di-µ3-4-aminophenylarsonato-tri-µ2-aqua-dipotassium] Data collection

**Table:**

Oxford Diffraction Gemini-S CCD-detector diffractometer	1834 independent reflections
Radiation source: Enhance (Mo) X-ray source	1710 reflections with *I* > 2σ(*I*)
Graphite monochromator	*R*_int_ = 0.020
Detector resolution: 16.077 pixels mm^-1^	θ_max_ = 29.3°, θ_min_ = 3.2°
ω scans	*h* = −32→17
Absorption correction: multi-scan (CrysAlis PRO; Rigaku OD, 2015)	*k* = −13→13
*T*_min_ = 0.650, *T*_max_ = 0.980	*l* = −9→10
2874 measured reflections	

### (I) Poly[di-µ3-4-aminophenylarsonato-tri-µ2-aqua-dipotassium] Refinement

**Table:**

Refinement on *F*^2^	Secondary atom site location: difference Fourier map
Least-squares matrix: full	Hydrogen site location: inferred from neighbouring sites
*R*[*F*^2^ > 2σ(*F*^2^)] = 0.030	H atoms treated by a mixture of independent and constrained refinement
*wR*(*F*^2^) = 0.125	*w* = 1/[σ^2^(*F*_o_^2^) + (0.0744*P*)^2^] where *P* = (*F*_o_^2^ + 2*F*_c_^2^)/3
*S* = 1.23	(Δ/σ)_max_ = 0.001
1834 reflections	Δρ_max_ = 0.50 e Å^−^^3^
145 parameters	Δρ_min_ = −0.84 e Å^−^^3^
8 restraints	Absolute structure: Flack (1983), 1281 Friedel pairs
Primary atom site location: structure-invariant direct methods	Absolute structure parameter: 0.03 (2)

### (I) Poly[di-µ3-4-aminophenylarsonato-tri-µ2-aqua-dipotassium] Special details

**Table:**

Geometry. Bond distances, angles etc. have been calculated using the rounded fractional coordinates. All su's are estimated from the variances of the (full) variance-covariance matrix. The cell esds are taken into account in the estimation of distances, angles and torsion angles
Refinement. Refinement of F^2^ against ALL reflections. The weighted R-factor wR and goodness of fit S are based on F^2^, conventional R-factors R are based on F, with F set to zero for negative F^2^. The threshold expression of F^2^ > 2sigma(F^2^) is used only for calculating R-factors(gt) etc. and is not relevant to the choice of reflections for refinement. R-factors based on F^2^ are statistically about twice as large as those based on F, and R- factors based on ALL data will be even larger.

### (I) Poly[di-µ3-4-aminophenylarsonato-tri-µ2-aqua-dipotassium] Fractional atomic coordinates and isotropic or equivalent isotropic displacement parameters (Å^2^)

**Table:**

	*x*	*y*	*z*	*U*_iso_*/*U*_eq_	
As1	0.60155 (2)	0.63813 (4)	0.55509 (10)	0.0164 (2)	
K1	0.50000	0.94523 (15)	0.5457 (3)	0.0224 (4)	
K2	0.50000	0.63621 (19)	0.2140 (3)	0.0279 (6)	
O1W	0.5845 (2)	1.0465 (4)	0.7396 (6)	0.0260 (12)	
O2W	0.50000	0.7951 (6)	−0.1067 (9)	0.0263 (19)	
O11	0.57103 (16)	0.7477 (4)	0.4358 (6)	0.0227 (12)	
O12	0.5720 (2)	0.4898 (4)	0.5138 (6)	0.0333 (16)	
O13	0.59063 (19)	0.6581 (4)	0.7630 (7)	0.0247 (14)	
N4	0.8483 (2)	0.6311 (5)	0.4372 (8)	0.0243 (17)	
C1	0.6778 (3)	0.6280 (5)	0.5052 (8)	0.0197 (16)	
C2	0.7027 (3)	0.7225 (5)	0.4072 (8)	0.0203 (16)	
C3	0.7583 (3)	0.7230 (5)	0.3828 (8)	0.0230 (17)	
C4	0.7918 (3)	0.6313 (5)	0.4615 (7)	0.0170 (17)	
C5	0.7669 (2)	0.5344 (4)	0.5574 (10)	0.0203 (14)	
C6	0.7104 (2)	0.5332 (5)	0.5803 (8)	0.0197 (16)	
H2	0.68080	0.78760	0.35650	0.0240*	
H3	0.77440	0.78640	0.31150	0.0270*	
H5	0.78880	0.46900	0.60720	0.0240*	
H6	0.69380	0.46780	0.64730	0.0240*	
H11W	0.608 (2)	0.998 (6)	0.795 (9)	0.0300*	
H12	0.585 (3)	0.450 (6)	0.425 (6)	0.0300*	
H12W	0.582 (3)	1.116 (4)	0.806 (8)	0.0300*	
H21W	0.525 (2)	0.781 (7)	−0.181 (7)	0.0300*	
H41	0.866 (3)	0.622 (5)	0.532 (5)	0.0240*	
H42	0.863 (3)	0.683 (6)	0.363 (7)	0.0240*	

### (I) Poly[di-µ3-4-aminophenylarsonato-tri-µ2-aqua-dipotassium] Atomic displacement parameters (Å^2^)

**Table:**

	*U*^11^	*U*^22^	*U*^33^	*U*^12^	*U*^13^	*U*^23^
As1	0.0178 (3)	0.0148 (3)	0.0167 (3)	−0.0020 (2)	0.0030 (3)	−0.0007 (3)
K1	0.0198 (7)	0.0217 (7)	0.0256 (8)	0.0000	0.0000	−0.0055 (9)
K2	0.0186 (9)	0.0387 (11)	0.0264 (10)	0.0000	0.0000	−0.0114 (8)
O1W	0.028 (2)	0.023 (2)	0.027 (2)	0.0021 (19)	−0.006 (2)	−0.001 (2)
O2W	0.018 (3)	0.027 (3)	0.034 (4)	0.0000	0.0000	−0.003 (3)
O11	0.019 (2)	0.026 (2)	0.023 (2)	−0.0026 (17)	0.0023 (18)	0.0053 (18)
O12	0.037 (3)	0.022 (2)	0.041 (3)	−0.0120 (19)	0.022 (2)	−0.014 (2)
O13	0.023 (2)	0.024 (2)	0.027 (3)	0.0033 (19)	0.008 (2)	0.004 (2)
N4	0.019 (3)	0.024 (3)	0.030 (3)	0.002 (2)	−0.001 (2)	0.002 (2)
C1	0.022 (3)	0.015 (2)	0.022 (3)	−0.001 (2)	0.001 (2)	−0.002 (2)
C2	0.022 (3)	0.017 (2)	0.022 (3)	0.007 (2)	−0.001 (2)	0.000 (2)
C3	0.026 (3)	0.018 (3)	0.025 (3)	−0.003 (2)	0.006 (2)	0.011 (3)
C4	0.025 (3)	0.021 (3)	0.005 (3)	0.005 (2)	0.005 (2)	−0.0027 (19)
C5	0.025 (2)	0.013 (2)	0.023 (3)	0.0061 (18)	−0.002 (3)	0.003 (3)
C6	0.024 (3)	0.016 (2)	0.019 (3)	0.0002 (19)	0.007 (2)	0.001 (2)

### (I) Poly[di-µ3-4-aminophenylarsonato-tri-µ2-aqua-dipotassium] Geometric parameters (Å, º)

**Table:**

K1—O1W	2.766 (5)	O2W—H21W	0.86 (5)
K1—O11	2.824 (4)	O2W—H21W^ii^	0.86 (5)
K1—O2W^i^	2.959 (7)	O12—H12	0.87 (6)
K1—O1W^ii^	2.766 (5)	N4—C4	1.389 (9)
K1—O11^ii^	2.824 (4)	N4—H41	0.86 (5)
K2—O2W	3.009 (7)	N4—H42	0.87 (6)
K2—O11	2.713 (5)	C1—C2	1.388 (8)
K2—O12^iii^	2.694 (5)	C1—C6	1.397 (8)
K2—O11^ii^	2.713 (5)	C2—C3	1.367 (10)
K2—O12^iv^	2.694 (5)	C3—C4	1.400 (9)
As1—O11	1.652 (4)	C4—C5	1.397 (8)
As1—O12	1.736 (4)	C5—C6	1.387 (7)
As1—O13	1.663 (5)	C2—H2	0.9500
As1—C1	1.900 (7)	C3—H3	0.9500
O1W—H11W	0.88 (6)	C5—H5	0.9500
O1W—H12W	0.89 (5)	C6—H6	0.9500
			
O11—As1—O12	108.9 (2)	K1—O1W—H12W	125 (5)
O11—As1—O13	113.3 (2)	H11W—O1W—H12W	103 (6)
O11—As1—C1	111.2 (2)	K1^v^—O2W—H21W	116 (5)
O12—As1—O13	103.2 (2)	H21W—O2W—H21W^ii^	91 (5)
O12—As1—C1	108.5 (2)	K1^v^—O2W—H21W^ii^	116 (5)
O13—As1—C1	111.4 (3)	K2—O2W—H21W	118 (4)
O1W—K1—O11	89.45 (13)	K2—O2W—H21W^ii^	118 (4)
O1W—K1—O2W^i^	82.66 (13)	K2^vi^—O12—H12	118 (4)
O1W—K1—O1W^ii^	96.08 (15)	As1—O12—H12	115 (4)
O1W—K1—O11^ii^	154.31 (14)	C4—N4—H41	112 (4)
O2W^i^—K1—O11	122.99 (14)	H41—N4—H42	116 (6)
O1W^ii^—K1—O11	154.31 (14)	C4—N4—H42	120 (5)
O11—K1—O11^ii^	75.52 (12)	As1—C1—C6	120.5 (4)
O1W^ii^—K1—O2W^i^	82.66 (13)	As1—C1—C2	120.1 (5)
O2W^i^—K1—O11^ii^	122.99 (14)	C2—C1—C6	119.1 (6)
O1W^ii^—K1—O11^ii^	89.45 (13)	C1—C2—C3	120.8 (6)
O2W—K2—O11	107.37 (14)	C2—C3—C4	120.9 (6)
O2W—K2—O12^iii^	77.45 (14)	C3—C4—C5	118.6 (6)
O2W—K2—O11^ii^	107.37 (14)	N4—C4—C3	121.2 (5)
O2W—K2—O12^iv^	77.45 (14)	N4—C4—C5	120.2 (5)
O11—K2—O12^iii^	175.18 (16)	C4—C5—C6	120.4 (5)
O11—K2—O11^ii^	79.20 (14)	C1—C6—C5	120.2 (5)
O11—K2—O12^iv^	99.61 (13)	C1—C2—H2	120.00
O11^ii^—K2—O12^iii^	99.61 (13)	C3—C2—H2	120.00
O12^iii^—K2—O12^iv^	81.18 (15)	C2—C3—H3	120.00
O11^ii^—K2—O12^iv^	175.18 (16)	C4—C3—H3	120.00
K1^v^—O2W—K2	99.6 (2)	C4—C5—H5	120.00
K1—O11—K2	96.75 (13)	C6—C5—H5	120.00
As1—O12—K2^vi^	126.6 (2)	C1—C6—H6	120.00
K1—O1W—H11W	122 (4)	C5—C6—H6	120.00
			
O12—As1—O11—K1	109.9 (3)	O11^ii^—K1—O11—K2	25.38 (14)
O12—As1—O11—K2	−6.6 (3)	O1W—K1—O11^ii^—K2	−81.4 (4)
O13—As1—O11—K1	−4.3 (3)	O11—K1—O11^ii^—K2	−25.38 (14)
O13—As1—O11—K2	−120.8 (2)	O2W—K2—O11—As1	−146.8 (2)
C1—As1—O11—K1	−130.6 (3)	O2W—K2—O11—K1	78.91 (15)
C1—As1—O11—K2	112.9 (2)	O11^ii^—K2—O11—As1	108.2 (2)
O11—As1—O12—K2^vi^	−103.8 (3)	O11^ii^—K2—O11—K1	−26.09 (14)
O13—As1—O12—K2^vi^	16.9 (3)	O12^iv^—K2—O11—As1	−67.1 (2)
C1—As1—O12—K2^vi^	135.1 (3)	O12^iv^—K2—O11—K1	158.66 (14)
O11—As1—C1—C2	12.4 (6)	O11—K2—O11^ii^—K1	26.09 (14)
O11—As1—C1—C6	−174.0 (5)	As1—C1—C2—C3	174.1 (5)
O12—As1—C1—C2	132.2 (5)	C6—C1—C2—C3	0.5 (9)
O12—As1—C1—C6	−54.3 (5)	As1—C1—C6—C5	−173.3 (5)
O13—As1—C1—C2	−114.9 (5)	C2—C1—C6—C5	0.3 (9)
O13—As1—C1—C6	58.6 (5)	C1—C2—C3—C4	−2.6 (9)
O1W—K1—O11—As1	61.8 (3)	C2—C3—C4—N4	179.9 (6)
O1W—K1—O11—K2	−175.68 (15)	C2—C3—C4—C5	3.8 (9)
O2W^i^—K1—O11—As1	142.7 (2)	N4—C4—C5—C6	−179.2 (6)
O1W^ii^—K1—O11—As1	−41.1 (5)	C3—C4—C5—C6	−3.0 (9)
O1W^ii^—K1—O11—K2	81.4 (4)	C4—C5—C6—C1	1.0 (9)
O11^ii^—K1—O11—As1	−97.1 (3)		

Symmetry codes: (i) −*x*+1, −*y*+2, *z*+1/2; (ii) −*x*+1, *y*, *z*; (iii) −*x*+1, −*y*+1, *z*−1/2; (iv) *x*, −*y*+1, *z*−1/2; (v) −*x*+1, −*y*+2, *z*−1/2; (vi) −*x*+1, −*y*+1, *z*+1/2.

### (I) Poly[di-µ3-4-aminophenylarsonato-tri-µ2-aqua-dipotassium] Hydrogen-bond geometry (Å, º)

**Table:**

*D*—H···*A*	*D*—H	H···*A*	*D*···*A*	*D*—H···*A*
O1*W*—H11*W*···N4^vii^	0.88 (6)	2.05 (6)	2.915 (7)	171 (5)
O1*W*—H12*W*···O11^viii^	0.89 (5)	1.77 (5)	2.660 (6)	175 (7)
O2*W*—H21*W*···O13^ix^	0.86 (5)	2.09 (6)	2.819 (6)	142 (6)
O12—H12···O13^iv^	0.87 (6)	1.70 (6)	2.538 (7)	160 (7)
N4—H41···O1*W*^x^	0.86 (5)	2.17 (5)	3.010 (7)	164 (5)
N4—H42···O13^xi^	0.87 (6)	2.15 (6)	2.984 (7)	160 (5)

Symmetry codes: (iv) *x*, −*y*+1, *z*−1/2; (vii) −*x*+3/2, −*y*+3/2, *z*+1/2; (viii) *x*, −*y*+2, *z*+1/2; (ix) *x*, *y*, *z*−1; (x) −*x*+3/2, *y*−1/2, *z*; (xi) −*x*+3/2, −*y*+3/2, *z*−1/2.

### (II) Poly[di-µ3-4-aminophenylarsonato-tri-µ2-aqua-dirubidium] Crystal data

**Table:**

[Rb_2_(C_6_H_7_AsNO_3_)_2_(H_2_O)_3_]	*F*(000) = 1272
*M**_r_* = 657.08	*D*_x_ = 2.105 Mg m^−^^3^
Orthorhombic, *C**m**c*2_1_	Mo *K*α radiation, λ = 0.71073 Å
Hall symbol: C 2c -2	Cell parameters from 1374 reflections
*a* = 24.4783 (19) Å	θ = 4.0–28.7°
*b* = 10.4577 (9) Å	µ = 7.94 mm^−^^1^
*c* = 8.0978 (7) Å	*T* = 200 K
*V* = 2072.9 (3) Å^3^	Prism, colourless
*Z* = 4	0.35 × 0.20 × 0.12 mm

### (II) Poly[di-µ3-4-aminophenylarsonato-tri-µ2-aqua-dirubidium] Data collection

**Table:**

Oxford Diffraction Gemini-S CCD-detector diffractometer	2093 independent reflections
Radiation source: Enhance (Mo) X-ray source	1899 reflections with *I* > 2σ(*I*)
Graphite monochromator	*R*_int_ = 0.026
Detector resolution: 16.077 pixels mm^-1^	θ_max_ = 29.0°, θ_min_ = 3.9°
ω scans	*h* = −33→15
Absorption correction: multi-scan (CrysAlis PRO; Rigaku OD, 2015)	*k* = −14→13
*T*_min_ = 0.375, *T*_max_ = 0.980	*l* = −10→10
3623 measured reflections	

### (II) Poly[di-µ3-4-aminophenylarsonato-tri-µ2-aqua-dirubidium] Refinement

**Table:**

Refinement on *F*^2^	Hydrogen site location: inferred from neighbouring sites
Least-squares matrix: full	H atoms treated by a mixture of independent and constrained refinement
*R*[*F*^2^ > 2σ(*F*^2^)] = 0.032	*w* = 1/[σ^2^(*F*_o_^2^) + (0.0392*P*)^2^] where *P* = (*F*_o_^2^ + 2*F*_c_^2^)/3
*wR*(*F*^2^) = 0.072	(Δ/σ)_max_ = 0.003
*S* = 1.03	Δρ_max_ = 0.70 e Å^−^^3^
2093 reflections	Δρ_min_ = −0.46 e Å^−^^3^
146 parameters	Extinction correction: SHELXL97, Fc^*^=kFc[1+0.001xFc^2^λ^3^/sin(2θ)]^-1/4^
8 restraints	Extinction coefficient: 0.00306 (19)
Primary atom site location: structure-invariant direct methods	Absolute structure: Flack (1983), 1309 Friedel pairs
Secondary atom site location: difference Fourier map	Absolute structure parameter: −0.008 (12)

### (II) Poly[di-µ3-4-aminophenylarsonato-tri-µ2-aqua-dirubidium] Special details

**Table:**

Geometry. Bond distances, angles etc. have been calculated using the rounded fractional coordinates. All su's are estimated from the variances of the (full) variance-covariance matrix. The cell esds are taken into account in the estimation of distances, angles and torsion angles
Refinement. Refinement of F^2^ against ALL reflections. The weighted R-factor wR and goodness of fit S are based on F^2^, conventional R-factors R are based on F, with F set to zero for negative F^2^. The threshold expression of F^2^ > 2sigma(F^2^) is used only for calculating R-factors(gt) etc. and is not relevant to the choice of reflections for refinement. R-factors based on F^2^ are statistically about twice as large as those based on F, and R- factors based on ALL data will be even larger.

### (II) Poly[di-µ3-4-aminophenylarsonato-tri-µ2-aqua-dirubidium] Fractional atomic coordinates and isotropic or equivalent isotropic displacement parameters (Å^2^)

**Table:**

	*x*	*y*	*z*	*U*_iso_*/*U*_eq_	
Rb1	0.50000	0.94747 (5)	0.57916 (11)	0.0264 (2)	
Rb2	0.50000	0.63207 (8)	0.24818 (10)	0.0376 (3)	
As1	0.60556 (2)	0.63552 (4)	0.58879 (6)	0.0200 (1)	
O1W	0.58793 (16)	1.0545 (3)	0.7791 (5)	0.0333 (11)	
O2W	0.50000	0.7864 (5)	−0.0920 (7)	0.0337 (17)	
O11	0.57535 (14)	0.7458 (3)	0.4753 (4)	0.0302 (11)	
O12	0.57765 (17)	0.4882 (3)	0.5436 (5)	0.0404 (13)	
O13	0.59344 (15)	0.6516 (3)	0.7898 (4)	0.0275 (11)	
N4	0.85111 (17)	0.6329 (4)	0.4734 (6)	0.0287 (16)	
C1	0.68166 (19)	0.6279 (4)	0.5443 (6)	0.0200 (14)	
C2	0.7060 (2)	0.7197 (5)	0.4430 (6)	0.0250 (16)	
C3	0.7614 (2)	0.7222 (5)	0.4197 (7)	0.0270 (17)	
C4	0.7946 (2)	0.6315 (4)	0.4982 (6)	0.0230 (16)	
C5	0.77044 (19)	0.5380 (4)	0.5957 (8)	0.0270 (14)	
C6	0.7146 (2)	0.5357 (4)	0.6178 (6)	0.0260 (16)	
H2	0.68380	0.78150	0.38940	0.0300*	
H3	0.77730	0.78530	0.35030	0.0320*	
H5	0.79260	0.47510	0.64760	0.0320*	
H6	0.69850	0.47060	0.68370	0.0310*	
H11W	0.6067 (19)	1.001 (4)	0.842 (6)	0.0370*	
H12	0.582 (2)	0.459 (5)	0.445 (3)	0.0370*	
H12W	0.589 (3)	1.127 (3)	0.833 (6)	0.0370*	
H21W	0.5290 (15)	0.741 (4)	−0.120 (7)	0.0370*	
H41	0.864 (2)	0.689 (4)	0.407 (6)	0.0300*	
H42	0.8680 (19)	0.613 (4)	0.566 (4)	0.0300*	

### (II) Poly[di-µ3-4-aminophenylarsonato-tri-µ2-aqua-dirubidium] Atomic displacement parameters (Å^2^)

**Table:**

	*U*^11^	*U*^22^	*U*^33^	*U*^12^	*U*^13^	*U*^23^
Rb1	0.0241 (3)	0.0282 (3)	0.0268 (3)	0.0000	0.0000	−0.0051 (4)
Rb2	0.0227 (4)	0.0597 (5)	0.0304 (4)	0.0000	0.0000	−0.0179 (4)
As1	0.0210 (2)	0.0183 (2)	0.0206 (2)	−0.0019 (2)	0.0036 (2)	−0.0007 (3)
O1W	0.032 (2)	0.0309 (19)	0.037 (2)	−0.0008 (16)	−0.0036 (19)	−0.0005 (18)
O2W	0.032 (3)	0.027 (3)	0.042 (3)	0.0000	0.0000	−0.006 (2)
O11	0.0206 (19)	0.037 (2)	0.033 (2)	−0.0005 (16)	−0.0041 (16)	0.0069 (16)
O12	0.046 (2)	0.0303 (18)	0.045 (3)	−0.0176 (17)	0.0261 (19)	−0.0182 (19)
O13	0.036 (2)	0.0257 (18)	0.0207 (18)	0.0053 (15)	0.0064 (16)	0.0031 (15)
N4	0.019 (2)	0.035 (3)	0.032 (3)	0.0024 (18)	0.0042 (19)	0.006 (2)
C1	0.019 (2)	0.021 (2)	0.020 (3)	0.0003 (17)	0.0009 (18)	−0.0017 (18)
C2	0.025 (3)	0.021 (2)	0.029 (3)	0.001 (2)	0.002 (2)	0.005 (2)
C3	0.023 (3)	0.025 (3)	0.033 (3)	0.001 (2)	0.006 (2)	0.009 (2)
C4	0.025 (3)	0.021 (2)	0.023 (3)	0.0018 (19)	0.004 (2)	−0.0043 (19)
C5	0.031 (2)	0.020 (2)	0.030 (3)	0.0078 (17)	0.003 (3)	0.003 (3)
C6	0.036 (3)	0.018 (2)	0.024 (3)	−0.0023 (19)	0.006 (2)	0.0040 (19)

### (II) Poly[di-µ3-4-aminophenylarsonato-tri-µ2-aqua-dirubidium] Geometric parameters (Å, º)

**Table:**

Rb1—O1W	2.917 (4)	O1W—H12W	0.88 (4)
Rb1—O11	2.925 (3)	O2W—H21W	0.88 (4)
Rb1—O2W^i^	3.151 (6)	O2W—H21W^iv^	0.88 (4)
Rb1—O1W^ii^	3.246 (4)	O12—H12	0.86 (3)
Rb1—O2W^iii^	3.109 (5)	N4—C4	1.398 (6)
Rb1—O1W^iv^	2.917 (4)	N4—H41	0.86 (5)
Rb1—O11^iv^	2.925 (3)	N4—H42	0.88 (4)
Rb1—O1W^v^	3.246 (4)	C1—C2	1.396 (7)
Rb2—O2W	3.193 (6)	C1—C6	1.391 (6)
Rb2—O11	2.863 (3)	C2—C3	1.369 (7)
Rb2—O12^vi^	2.818 (4)	C3—C4	1.402 (7)
Rb2—O11^iv^	2.863 (3)	C4—C5	1.389 (7)
Rb2—O12^vii^	2.818 (4)	C5—C6	1.379 (7)
As1—O11	1.650 (3)	C2—H2	0.9500
As1—O12	1.725 (3)	C3—H3	0.9500
As1—O13	1.663 (3)	C5—H5	0.9500
As1—C1	1.899 (5)	C6—H6	0.9500
O1W—H11W	0.89 (5)		
			
O1W—Rb1—O11	88.34 (10)	Rb1—O1W—Rb1^iii^	85.63 (10)
O1W—Rb1—O2W^i^	74.69 (10)	Rb1^viii^—O2W—Rb2	178.05 (19)
O1W—Rb1—O1W^ii^	155.13 (9)	Rb1^ii^—O2W—Rb2	93.89 (15)
O1W—Rb1—O2W^iii^	84.49 (10)	Rb1^viii^—O2W—Rb1^ii^	84.16 (13)
O1W—Rb1—O1W^iv^	95.12 (11)	Rb1—O11—Rb2	94.47 (10)
O1W—Rb1—O11^iv^	154.37 (10)	Rb1—O11—As1	128.79 (16)
O1W—Rb1—O1W^v^	85.91 (10)	Rb2—O11—As1	110.86 (15)
O2W^i^—Rb1—O11	81.81 (10)	Rb2^ix^—O12—As1	122.77 (19)
O1W^ii^—Rb1—O11	101.44 (9)	Rb1^iii^—O1W—H12W	70 (4)
O2W^iii^—Rb1—O11	121.14 (10)	Rb1—O1W—H12W	129 (5)
O1W^iv^—Rb1—O11	154.37 (10)	H11W—O1W—H12W	104 (5)
O11—Rb1—O11^iv^	78.18 (9)	Rb1—O1W—H11W	117 (3)
O1W^v^—Rb1—O11	50.37 (9)	Rb1^iii^—O1W—H11W	85 (3)
O1W^ii^—Rb1—O2W^i^	128.99 (8)	Rb2—O2W—H21W^iv^	87 (4)
O2W^i^—Rb1—O2W^iii^	148.79 (14)	Rb1^viii^—O2W—H21W	94 (4)
O1W^iv^—Rb1—O2W^i^	74.69 (10)	Rb2—O2W—H21W	87 (4)
O2W^i^—Rb1—O11^iv^	81.81 (10)	H21W—O2W—H21W^iv^	107 (4)
O1W^v^—Rb1—O2W^i^	128.99 (8)	Rb1^viii^—O2W—H21W^iv^	94 (4)
O1W^ii^—Rb1—O2W^iii^	70.85 (9)	Rb1^ii^—O2W—H21W	127 (3)
O1W^ii^—Rb1—O1W^iv^	85.91 (10)	Rb1^ii^—O2W—H21W^iv^	127 (3)
O1W^ii^—Rb1—O11^iv^	50.37 (9)	Rb2^ix^—O12—H12	118 (3)
O1W^ii^—Rb1—O1W^v^	83.07 (10)	As1—O12—H12	118 (3)
O1W^iv^—Rb1—O2W^iii^	84.49 (10)	C4—N4—H41	118 (3)
O2W^iii^—Rb1—O11^iv^	121.14 (10)	C4—N4—H42	110 (3)
O1W^v^—Rb1—O2W^iii^	70.85 (9)	H41—N4—H42	122 (4)
O1W^iv^—Rb1—O11^iv^	88.34 (10)	C2—C1—C6	118.7 (4)
O1W^iv^—Rb1—O1W^v^	155.13 (9)	As1—C1—C2	120.1 (3)
O1W^v^—Rb1—O11^iv^	101.44 (9)	As1—C1—C6	121.1 (3)
O2W—Rb2—O11	110.13 (10)	C1—C2—C3	121.1 (5)
O2W—Rb2—O12^vi^	73.64 (10)	C2—C3—C4	119.9 (5)
O2W—Rb2—O11^iv^	110.13 (10)	N4—C4—C5	120.7 (4)
O2W—Rb2—O12^vii^	73.64 (10)	N4—C4—C3	120.1 (4)
O11—Rb2—O12^vi^	176.01 (11)	C3—C4—C5	119.2 (4)
O11—Rb2—O11^iv^	80.20 (10)	C4—C5—C6	120.5 (4)
O11—Rb2—O12^vii^	97.38 (11)	C1—C6—C5	120.5 (4)
O11^iv^—Rb2—O12^vi^	97.38 (11)	C1—C2—H2	119.00
O12^vi^—Rb2—O12^vii^	84.84 (12)	C3—C2—H2	119.00
O11^iv^—Rb2—O12^vii^	176.01 (11)	C2—C3—H3	120.00
O11—As1—O12	109.20 (17)	C4—C3—H3	120.00
O11—As1—O13	113.24 (16)	C4—C5—H5	120.00
O11—As1—C1	111.30 (18)	C6—C5—H5	120.00
O12—As1—O13	103.14 (18)	C1—C6—H6	120.00
O12—As1—C1	108.11 (19)	C5—C6—H6	120.00
O13—As1—C1	111.40 (19)		
			
O11—Rb1—O1W—Rb1^iii^	−128.95 (9)	O13—As1—O11—Rb1	−4.5 (3)
O1W—Rb1—O11—Rb2	−175.57 (10)	O13—As1—O11—Rb2	−118.92 (17)
O1W—Rb1—O11—As1	63.0 (2)	C1—As1—O11—Rb1	−130.9 (2)
O2W^i^—Rb1—O11—As1	−11.8 (2)	C1—As1—O11—Rb2	114.66 (18)
O1W^ii^—Rb1—O11—Rb2	−18.62 (11)	O11—As1—O12—Rb2^ix^	−101.6 (2)
O1W^ii^—Rb1—O11—As1	−140.0 (2)	O13—As1—O12—Rb2^ix^	19.1 (2)
O2W^iii^—Rb1—O11—As1	145.56 (19)	C1—As1—O12—Rb2^ix^	137.2 (2)
O1W^iv^—Rb1—O11—Rb2	86.1 (2)	O11—As1—C1—C2	7.8 (4)
O1W^iv^—Rb1—O11—As1	−35.4 (4)	O11—As1—C1—C6	−175.6 (4)
O11^iv^—Rb1—O11—Rb2	26.37 (9)	O12—As1—C1—C2	127.7 (4)
O11^iv^—Rb1—O11—As1	−95.1 (2)	O12—As1—C1—C6	−55.6 (4)
O1W^v^—Rb1—O11—Rb2	−89.51 (13)	O13—As1—C1—C2	−119.7 (4)
O1W^v^—Rb1—O11—As1	149.1 (3)	O13—As1—C1—C6	57.0 (4)
O1W—Rb1—O11^iv^—Rb2	−86.1 (2)	As1—C1—C2—C3	174.8 (4)
O11—Rb1—O11^iv^—Rb2	−26.37 (9)	C6—C1—C2—C3	−2.0 (7)
O2W—Rb2—O11—Rb1	81.20 (11)	As1—C1—C6—C5	−174.4 (4)
O2W—Rb2—O11—As1	−144.19 (15)	C2—C1—C6—C5	2.4 (7)
O11^iv^—Rb2—O11—Rb1	−26.79 (9)	C1—C2—C3—C4	0.0 (8)
O11^iv^—Rb2—O11—As1	107.83 (17)	C2—C3—C4—N4	179.3 (5)
O12^vii^—Rb2—O11—Rb1	156.43 (10)	C2—C3—C4—C5	1.6 (8)
O12^vii^—Rb2—O11—As1	−68.96 (17)	N4—C4—C5—C6	−179.0 (5)
O11—Rb2—O11^iv^—Rb1	26.79 (9)	C3—C4—C5—C6	−1.3 (8)
O12—As1—O11—Rb1	109.8 (2)	C4—C5—C6—C1	−0.8 (8)
O12—As1—O11—Rb2	−4.6 (2)		

Symmetry codes: (i) *x*, *y*, *z*+1; (ii) −*x*+1, −*y*+2, *z*−1/2; (iii) −*x*+1, −*y*+2, *z*+1/2; (iv) −*x*+1, *y*, *z*; (v) *x*, −*y*+2, *z*−1/2; (vi) −*x*+1, −*y*+1, *z*−1/2; (vii) *x*, −*y*+1, *z*−1/2; (viii) *x*, *y*, *z*−1; (ix) −*x*+1, −*y*+1, *z*+1/2.

### (II) Poly[di-µ3-4-aminophenylarsonato-tri-µ2-aqua-dirubidium] Hydrogen-bond geometry (Å, º)

**Table:**

*D*—H···*A*	*D*—H	H···*A*	*D*···*A*	*D*—H···*A*
O1*W*—H11*W*···N4^x^	0.89 (5)	2.04 (4)	2.923 (6)	176 (5)
O2*W*—H21*W*···O13^viii^	0.88 (4)	1.97 (4)	2.852 (5)	173 (5)
O12—H12···O13^vii^	0.86 (3)	1.73 (4)	2.552 (5)	158 (5)
N4—H41···O13^xi^	0.86 (5)	2.18 (4)	3.022 (5)	167 (4)
N4—H42···O1*W*^xii^	0.88 (4)	2.13 (4)	3.005 (6)	176 (4)

Symmetry codes: (vii) *x*, −*y*+1, *z*−1/2; (viii) *x*, *y*, *z*−1; (x) −*x*+3/2, −*y*+3/2, *z*+1/2; (xi) −*x*+3/2, −*y*+3/2, *z*−1/2; (xii) −*x*+3/2, *y*−1/2, *z*.

### (III) Poly[di-µ3-4-aminophenylarsonato-tri-µ2-aqua-dicaesium] Crystal data

**Table:**

[Cs_2_(C_6_H_7_AsNO_3_)_2_(H_2_O)_3_]	*F*(000) = 1416
*M**_r_* = 751.96	*D*_x_ = 2.311 Mg m^−^^3^
Orthorhombic, *C**m**c*2_1_	Mo *K*α radiation, λ = 0.71073 Å
Hall symbol: C 2c -2	Cell parameters from 1999 reflections
*a* = 24.650 (3) Å	θ = 4.0–28.9°
*b* = 10.4373 (9) Å	µ = 6.46 mm^−^^1^
*c* = 8.3992 (7) Å	*T* = 200 K
*V* = 2160.9 (4) Å^3^	Prism, colourless
*Z* = 4	0.40 × 0.22 × 0.10 mm

### (III) Poly[di-µ3-4-aminophenylarsonato-tri-µ2-aqua-dicaesium] Data collection

**Table:**

Oxford Diffraction Gemini-S CCD-detector diffractometer	1883 independent reflections
Radiation source: Enhance Mo X-ray source	1787 reflections with *I* > 2σ(*I*)
Graphite monochromator	*R*_int_ = 0.030
Detector resolution: 16.077 pixels mm^-1^	θ_max_ = 29.3°, θ_min_ = 3.2°
ω scans	*h* = −33→29
Absorption correction: multi-scan (CrysAlis PRO; Rigaku OD, 2015)	*k* = −14→13
*T*_min_ = 0.217, *T*_max_ = 0.980	*l* = −11→6
4941 measured reflections	

### (III) Poly[di-µ3-4-aminophenylarsonato-tri-µ2-aqua-dicaesium] Refinement

**Table:**

Refinement on *F*^2^	Secondary atom site location: difference Fourier map
Least-squares matrix: full	Hydrogen site location: inferred from neighbouring sites
*R*[*F*^2^ > 2σ(*F*^2^)] = 0.041	H atoms treated by a mixture of independent and constrained refinement
*wR*(*F*^2^) = 0.160	*w* = 1/[σ^2^(*F*_o_^2^) + (0.1178*P*)^2^] where *P* = (*F*_o_^2^ + 2*F*_c_^2^)/3
*S* = 1.18	(Δ/σ)_max_ = 0.001
1883 reflections	Δρ_max_ = 1.69 e Å^−^^3^
145 parameters	Δρ_min_ = −1.00 e Å^−^^3^
8 restraints	Absolute structure: Flack (1983), 1405 Friedel pairs
Primary atom site location: structure-invariant direct methods	Absolute structure parameter: 0.10 (4)

### (III) Poly[di-µ3-4-aminophenylarsonato-tri-µ2-aqua-dicaesium] Special details

**Table:**

Geometry. Bond distances, angles etc. have been calculated using the rounded fractional coordinates. All su's are estimated from the variances of the (full) variance-covariance matrix. The cell esds are taken into account in the estimation of distances, angles and torsion angles
Refinement. Refinement of F^2^ against ALL reflections. The weighted R-factor wR and goodness of fit S are based on F^2^, conventional R-factors R are based on F, with F set to zero for negative F^2^. The threshold expression of F^2^ > 2sigma(F^2^) is used only for calculating R-factors(gt) etc. and is not relevant to the choice of reflections for refinement. R-factors based on F^2^ are statistically about twice as large as those based on F, and R- factors based on ALL data will be even larger.

### (III) Poly[di-µ3-4-aminophenylarsonato-tri-µ2-aqua-dicaesium] Fractional atomic coordinates and isotropic or equivalent isotropic displacement parameters (Å^2^)

**Table:**

	*x*	*y*	*z*	*U*_iso_*/*U*_eq_	
Cs1	0.50000	0.95299 (7)	0.67229 (13)	0.0217 (2)	
Cs2	0.50000	0.62982 (10)	0.99928 (13)	0.0311 (3)	
As1	0.61041 (4)	0.63299 (7)	0.65867 (12)	0.0166 (3)	
O1W	0.5936 (4)	1.0619 (7)	0.4690 (11)	0.026 (3)	
O2W	0.50000	0.7680 (11)	0.3558 (16)	0.027 (3)	
O11	0.5799 (3)	0.7443 (8)	0.7661 (11)	0.028 (3)	
O12	0.5841 (4)	0.4827 (8)	0.7086 (11)	0.036 (3)	
O13	0.5962 (3)	0.6457 (7)	0.4668 (11)	0.024 (2)	
N4	0.8542 (4)	0.6351 (9)	0.7686 (14)	0.025 (3)	
C1	0.6861 (5)	0.6283 (7)	0.6996 (13)	0.016 (3)	
C2	0.7088 (5)	0.7189 (9)	0.7999 (14)	0.022 (3)	
C3	0.7634 (5)	0.7201 (9)	0.8256 (18)	0.025 (3)	
C4	0.7983 (5)	0.6344 (9)	0.7490 (14)	0.022 (3)	
C5	0.7752 (5)	0.5399 (9)	0.6487 (19)	0.031 (4)	
C6	0.7202 (5)	0.5380 (8)	0.6236 (15)	0.023 (3)	
H2	0.68620	0.78020	0.85100	0.0260*	
H3	0.77820	0.78100	0.89760	0.0300*	
H5	0.79770	0.47800	0.59880	0.0370*	
H6	0.70480	0.47550	0.55480	0.0270*	
H11W	0.624 (3)	1.039 (12)	0.420 (15)	0.0340*	
H12	0.578 (7)	0.493 (15)	0.811 (5)	0.0340*	
H12W	0.589 (4)	1.126 (12)	0.400 (12)	0.0340*	
H21W	0.529 (4)	0.773 (13)	0.418 (16)	0.0340*	
H41	0.865 (6)	0.602 (12)	0.685 (18)	0.0270*	
H42	0.870 (6)	0.706 (11)	0.81 (2)	0.0270*	

### (III) Poly[di-µ3-4-aminophenylarsonato-tri-µ2-aqua-dicaesium] Atomic displacement parameters (Å^2^)

**Table:**

	*U*^11^	*U*^22^	*U*^33^	*U*^12^	*U*^13^	*U*^23^
Cs1	0.0185 (4)	0.0261 (4)	0.0204 (4)	0.0000	0.0000	0.0024 (4)
Cs2	0.0174 (5)	0.0525 (6)	0.0233 (5)	0.0000	0.0000	0.0136 (5)
As1	0.0146 (5)	0.0182 (4)	0.0169 (5)	−0.0016 (3)	−0.0029 (5)	0.0004 (4)
O1W	0.017 (4)	0.035 (4)	0.026 (5)	0.000 (3)	0.009 (4)	0.002 (4)
O2W	0.020 (6)	0.031 (5)	0.030 (7)	0.0000	0.0000	0.002 (5)
O11	0.020 (4)	0.032 (4)	0.031 (5)	−0.002 (3)	0.007 (4)	−0.005 (3)
O12	0.042 (5)	0.033 (4)	0.032 (5)	−0.021 (4)	−0.024 (4)	0.007 (4)
O13	0.019 (4)	0.029 (4)	0.025 (4)	0.005 (3)	0.000 (4)	0.001 (3)
N4	0.020 (5)	0.026 (4)	0.030 (5)	0.003 (3)	−0.015 (5)	−0.004 (4)
C1	0.019 (5)	0.015 (4)	0.014 (4)	0.000 (3)	−0.004 (4)	0.001 (3)
C2	0.027 (6)	0.020 (4)	0.019 (5)	0.006 (4)	−0.009 (4)	−0.011 (4)
C3	0.022 (5)	0.024 (4)	0.030 (6)	0.003 (4)	0.006 (6)	−0.002 (5)
C4	0.021 (6)	0.021 (4)	0.023 (6)	0.004 (4)	0.002 (5)	0.006 (4)
C5	0.028 (6)	0.024 (4)	0.041 (8)	0.006 (4)	−0.005 (6)	−0.013 (5)
C6	0.020 (5)	0.020 (4)	0.029 (6)	−0.003 (3)	−0.005 (5)	−0.002 (4)

### (III) Poly[di-µ3-4-aminophenylarsonato-tri-µ2-aqua-dicaesium] Geometric parameters (Å, º)

**Table:**

Cs1—O1W	3.087 (9)	O1W—H12W	0.89 (12)
Cs1—O2W	3.286 (13)	O2W—H21W	0.89 (12)
Cs1—O11	3.040 (8)	O2W—H21W^ii^	0.89 (12)
Cs1—O1W^i^	3.400 (10)	O12—H12	0.88 (5)
Cs1—O2W^i^	3.295 (12)	N4—C4	1.388 (16)
Cs1—O1W^ii^	3.087 (9)	N4—H41	0.83 (14)
Cs1—O11^ii^	3.040 (8)	N4—H42	0.91 (14)
Cs1—O1W^iii^	3.400 (10)	C1—C2	1.385 (15)
Cs2—O11	3.024 (8)	C1—C6	1.415 (15)
Cs2—O2W^iv^	3.324 (13)	C2—C3	1.363 (17)
Cs2—O12^v^	2.961 (9)	C3—C4	1.398 (16)
Cs2—O11^ii^	3.024 (8)	C4—C5	1.417 (17)
Cs2—O12^vi^	2.961 (9)	C5—C6	1.372 (18)
As1—O11	1.652 (9)	C2—H2	0.9500
As1—O12	1.748 (9)	C3—H3	0.9500
As1—O13	1.655 (9)	C5—H5	0.9500
As1—C1	1.898 (12)	C6—H6	0.9500
O1W—H11W	0.89 (9)		
			
O1W—Cs1—O2W	76.6 (2)	Cs1—O1W—Cs1^vii^	83.2 (2)
O1W—Cs1—O11	85.6 (2)	Cs1—O2W—Cs2^viii^	169.7 (4)
O1W—Cs1—O1W^i^	158.4 (2)	Cs1—O2W—Cs1^vii^	81.9 (3)
O1W—Cs1—O2W^i^	86.2 (2)	Cs1^vii^—O2W—Cs2^viii^	87.8 (3)
O1W—Cs1—O1W^ii^	96.7 (2)	Cs1—O11—Cs2	91.7 (2)
O1W—Cs1—O11^ii^	153.0 (2)	Cs1—O11—As1	131.1 (4)
O1W—Cs1—O1W^iii^	85.1 (2)	Cs2—O11—As1	111.9 (4)
O2W—Cs1—O11	77.8 (2)	Cs2^ix^—O12—As1	118.2 (4)
O1W^i^—Cs1—O2W	124.49 (19)	Cs1^vii^—O1W—H12W	58 (7)
O2W—Cs1—O2W^i^	153.9 (3)	Cs1—O1W—H12W	123 (7)
O1W^ii^—Cs1—O2W	76.6 (2)	H11W—O1W—H12W	91 (11)
O2W—Cs1—O11^ii^	77.8 (2)	Cs1—O1W—H11W	142 (8)
O1W^iii^—Cs1—O2W	124.49 (19)	Cs1^vii^—O1W—H11W	103 (8)
O1W^i^—Cs1—O11	102.5 (2)	Cs1—O2W—H21W^ii^	59 (8)
O2W^i^—Cs1—O11	120.8 (2)	Cs2^viii^—O2W—H21W	124 (7)
O1W^ii^—Cs1—O11	153.0 (2)	Cs1—O2W—H21W	59 (8)
O11—Cs1—O11^ii^	80.8 (2)	H21W—O2W—H21W^ii^	107 (11)
O1W^iii^—Cs1—O11	48.5 (2)	Cs2^viii^—O2W—H21W^ii^	124 (8)
O1W^i^—Cs1—O2W^i^	72.4 (2)	Cs1^vii^—O2W—H21W	103 (8)
O1W^i^—Cs1—O1W^ii^	85.1 (2)	Cs1^vii^—O2W—H21W^ii^	103 (9)
O1W^i^—Cs1—O11^ii^	48.5 (2)	Cs2^ix^—O12—H12	121 (11)
O1W^i^—Cs1—O1W^iii^	85.5 (2)	As1—O12—H12	101 (10)
O1W^ii^—Cs1—O2W^i^	86.2 (2)	C4—N4—H41	103 (10)
O2W^i^—Cs1—O11^ii^	120.8 (2)	C4—N4—H42	119 (8)
O1W^iii^—Cs1—O2W^i^	72.4 (2)	H41—N4—H42	122 (13)
O1W^ii^—Cs1—O11^ii^	85.6 (2)	C2—C1—C6	119.3 (11)
O1W^ii^—Cs1—O1W^iii^	158.4 (2)	As1—C1—C2	119.3 (8)
O1W^iii^—Cs1—O11^ii^	102.5 (2)	As1—C1—C6	121.3 (8)
O2W^iv^—Cs2—O11	114.3 (2)	C1—C2—C3	120.1 (11)
O11—Cs2—O12^v^	175.8 (2)	C2—C3—C4	121.9 (11)
O11—Cs2—O11^ii^	81.3 (2)	N4—C4—C5	118.3 (10)
O11—Cs2—O12^vi^	94.9 (2)	N4—C4—C3	123.6 (10)
O2W^iv^—Cs2—O12^v^	68.7 (2)	C3—C4—C5	118.2 (11)
O2W^iv^—Cs2—O11^ii^	114.3 (2)	C4—C5—C6	119.9 (11)
O2W^iv^—Cs2—O12^vi^	68.7 (2)	C1—C6—C5	120.5 (10)
O11^ii^—Cs2—O12^v^	94.9 (2)	C1—C2—H2	120.00
O12^v^—Cs2—O12^vi^	88.9 (3)	C3—C2—H2	120.00
O11^ii^—Cs2—O12^vi^	175.8 (2)	C2—C3—H3	119.00
O11—As1—O12	109.3 (4)	C4—C3—H3	119.00
O11—As1—O13	112.3 (4)	C4—C5—H5	120.00
O11—As1—C1	111.5 (4)	C6—C5—H5	120.00
O12—As1—O13	103.1 (4)	C1—C6—H6	120.00
O12—As1—C1	107.3 (4)	C5—C6—H6	120.00
O13—As1—C1	112.8 (4)		
			
O11—Cs1—O1W—Cs1^vii^	126.4 (2)	O13—As1—O11—Cs1	3.5 (6)
O1W—Cs1—O11—Cs2	175.0 (2)	O13—As1—O11—Cs2	116.5 (4)
O1W—Cs1—O11—As1	−63.7 (5)	C1—As1—O11—Cs1	131.1 (5)
O2W—Cs1—O11—Cs2	−107.8 (2)	C1—As1—O11—Cs2	−115.8 (4)
O2W—Cs1—O11—As1	13.5 (5)	O11—As1—O12—Cs2^ix^	98.9 (5)
O1W^i^—Cs1—O11—Cs2	15.3 (3)	O13—As1—O12—Cs2^ix^	−20.8 (5)
O1W^i^—Cs1—O11—As1	136.6 (5)	C1—As1—O12—Cs2^ix^	−140.0 (5)
O2W^i^—Cs1—O11—As1	−146.6 (5)	O11—As1—C1—C2	−4.7 (10)
O1W^ii^—Cs1—O11—Cs2	−88.9 (5)	O11—As1—C1—C6	178.4 (8)
O1W^ii^—Cs1—O11—As1	32.4 (9)	O12—As1—C1—C2	−124.4 (8)
O11^ii^—Cs1—O11—Cs2	−28.4 (2)	O12—As1—C1—C6	58.7 (9)
O11^ii^—Cs1—O11—As1	93.0 (5)	O13—As1—C1—C2	122.7 (8)
O1W^iii^—Cs1—O11—Cs2	87.6 (3)	O13—As1—C1—C6	−54.2 (9)
O1W^iii^—Cs1—O11—As1	−151.1 (6)	As1—C1—C2—C3	−177.4 (9)
O1W—Cs1—O11^ii^—Cs2	88.9 (5)	C6—C1—C2—C3	−0.5 (16)
O11—Cs1—O11^ii^—Cs2	28.4 (2)	As1—C1—C6—C5	176.9 (9)
O2W^iv^—Cs2—O11—As1	139.6 (4)	C2—C1—C6—C5	0.0 (16)
O11^ii^—Cs2—O11—Cs1	28.5 (2)	C1—C2—C3—C4	2.2 (18)
O11^ii^—Cs2—O11—As1	−107.6 (4)	C2—C3—C4—N4	178.1 (11)
O12^vi^—Cs2—O11—Cs1	−153.2 (2)	C2—C3—C4—C5	−3.4 (18)
O12^vi^—Cs2—O11—As1	70.7 (4)	N4—C4—C5—C6	−178.5 (11)
O11—Cs2—O11^ii^—Cs1	−28.5 (2)	C3—C4—C5—C6	2.9 (18)
O12—As1—O11—Cs1	−110.3 (5)	C4—C5—C6—C1	−1.2 (18)
O12—As1—O11—Cs2	2.7 (5)		

Symmetry codes: (i) −*x*+1, −*y*+2, *z*+1/2; (ii) −*x*+1, *y*, *z*; (iii) *x*, −*y*+2, *z*+1/2; (iv) *x*, *y*, *z*+1; (v) −*x*+1, −*y*+1, *z*+1/2; (vi) *x*, −*y*+1, *z*+1/2; (vii) −*x*+1, −*y*+2, *z*−1/2; (viii) *x*, *y*, *z*−1; (ix) −*x*+1, −*y*+1, *z*−1/2.

### (III) Poly[di-µ3-4-aminophenylarsonato-tri-µ2-aqua-dicaesium] Hydrogen-bond geometry (Å, º)

**Table:**

*D*—H···*A*	*D*—H	H···*A*	*D*···*A*	*D*—H···*A*
O1*W*—H11*W*···N4^x^	0.89 (9)	2.28 (13)	2.952 (13)	132 (10)
O2*W*—H21*W*···O13	0.89 (12)	2.16 (12)	2.850 (10)	134 (12)
O12—H12···O13^vi^	0.88 (5)	2.00 (12)	2.567 (13)	121 (12)
N4—H41···O1*W*^xi^	0.83 (14)	2.12 (15)	2.928 (15)	164 (9)
N4—H42···O13^xii^	0.91 (14)	2.20 (14)	3.082 (13)	166 (15)

Symmetry codes: (vi) *x*, −*y*+1, *z*+1/2; (x) −*x*+3/2, −*y*+3/2, *z*−1/2; (xi) −*x*+3/2, *y*−1/2, *z*; (xii) −*x*+3/2, −*y*+3/2, *z*+1/2.
